# Customized Titanium Plates for Preventing Mandibular Fractures in Lower Third Molar Extractions

**DOI:** 10.3390/jfb16020064

**Published:** 2025-02-13

**Authors:** Cezara Andreea Onică, Costin Iulian Lupu, Elena-Raluca Baciu, Gabriela Luminița Gelețu, Alice Murariu, Dana Gabriela Budală, Ionuț Luchian, Neculai Onică

**Affiliations:** 1 Department of Surgery, Faculty of Dental Medicine, University of Medicine and Pharmacy “Grigore T. Popa”, 700115 Iasi, Romania; cezara-andreea.onica@umfiasi.ro (C.A.O.); gabriela.geletu@umfiasi.ro (G.L.G.); alice.murariu@umfiasi.ro (A.M.); onica_neculai@d.umfiasi.ro (N.O.); 2 Department of Implantology, Removable Dentures, Dental Technology, Faculty of Dental Medicine, University of Medicine and Pharmacy “Grigore T. Popa”, 700115 Iasi, Romania; elena.baciu@umfiasi.ro (E.-R.B.); dana-gabriela.bosinceanu@umfiasi.ro (D.G.B.); 3 Department of Periodontology, Faculty of Dental Medicine, University of Medicine and Pharmacy “Grigore T. Popa”, 700115 Iasi, Romania

**Keywords:** third molar extraction, facial scanner, customized titanium plate, 3D printing

## Abstract

The extraction of deeply impacted lower third molars is a common yet challenging surgical procedure associated with complications such as mandibular fractures, pain, and swelling. This study evaluated the effectiveness of customized 3D-printed titanium plates in reducing the risk of intraoperative iatrogenic mandibular fractures. This innovative approach aims to improve surgical outcomes, enhance patient safety, and boost confidence for both surgeons and patients. Eighteen patients with Pell and Gregory class II/IIIC impacted lower third molars underwent preoperative CBCT scans, which facilitated the design and fabrication of customized plates and drilling guides. The surgical procedure involved incision, flap elevation, precise plate placement, osteotomy, odontotomy, extraction, and the postoperative assessment of pain, swelling, trismus, and anxiety using validated scales and facial scanning. The results show that customized titanium plates successfully prevented mandibular fractures in all cases. Although initial postoperative discomfort, including swelling, trismus, and pain, was observed, significant improvements occurred within one week. This technique provided structural reinforcement during surgery and healing without adverse events or fractures. Customized 3D-printed titanium plates represent a safe and effective solution for minimizing mandibular fractures, offering promising improvements in surgical outcomes.

## 1. Introduction

Traditionally, surgeons relied on their “best estimate” during conventional surgeries [1]. However, advancements in computer-aided design and computer-assisted manufacturing (CAD/CAM) have transformed surgical procedures by enabling the production of custom-made titanium meshes, plates, and implants based on an individual’s virtual surgical plan [2,3,4,5,6,7]. These innovations have revolutionized the field, allowing for highly precise and patient-specific solutions. Recently, custom titanium plates have become a superior alternative to pre-formed and other custom plate options [8]. During preoperative virtual planning (VP) sessions, surgeons determine the specific details of the plates, including the number, angle, and location of screw holes, as well as the contour and thickness of the plates [9]. Advances in manufacturing technologies such as conventional milling and rapid production techniques, like selective laser melting (SLM), electron beam melting (EBM), and fused deposition modeling (FDM), have enhanced the precision, efficiency, and customization of these solutions. These techniques allow for the fabrication of complex geometries with excellent mechanical properties, optimizing outcomes for patient-specific surgical applications [5,6,7].

In 1975, William Harry Archer defined an impacted tooth as one that is either completely or partially unerupted and is obstructed by another tooth, bone, or soft tissue, preventing further eruption [10]. Impacted mandibular third molars are frequently encountered in clinical practice and are associated with various complications, including caries, pain, swelling, paresthesia, periodontal pockets, pericoronitis, and pathological conditions such as cysts and tumors. The removal of these teeth is a common practice; however, this procedure can lead to complications influenced by patient factors (age, gender, health status), local anatomical considerations (tooth angulation, degree of impaction), and surgical variables such as operator experience and the type of equipment used. Common complications include hemorrhage, dry socket, infection, and nerve injury [11,12,13,14]. Rare but severe complications, such as trismus, iatrogenic damage to adjacent teeth, and iatrogenic mandibular fractures with an incidence of 0.0034% to 0.0075%, may also occur. These fractures are particularly distressing and might occur intraoperatively or postoperatively, highlighting the need for strategies to mitigate such risks during the surgical extraction of impacted mandibular third molars [15,16].

Postoperative fractures are more common than intraoperative ones, with 57% occurring during the second and third weeks after surgery. This increased risk is attributed to the significant bone removal required for extracting bony impacted wisdom teeth, emphasizing the importance of preserving full dentition. Additionally, the left side of the patient’s jaw is more prone to acute fractures. To minimize this risk, Woldenberg et al. [17] recommend specific measures for both doctors and patients. For doctors, it is essential to conduct thorough analyses of cone beam computed tomography (CBCT) scans, adopt minimally invasive approaches, and inform patients about the potential risk of fractures. Recent advances in third molar surgery have significantly contributed to reducing intraoperative difficulty, postoperative morbidity, and the risk of fractures [18].

Our study aimed to investigate the introduction of customized 3D-printed titanium plates (CTPs) as an additional surgical step to significantly reduce the risk of iatrogenic mandibular fractures, thereby enhancing both doctor and patient confidence. For this purpose, we formulated the null hypothesis that the new approach does not significantly reduce the risk of iatrogenic mandibular fractures and does not have a significant impact on anxiety, pain, swelling, trismus, or surgery time.

## 2. Materials and Methods

### 2.1. Study Design and Criteria for Patient Selection

This single-center study was conducted between November 2023 and May 2024 in accordance with the ethical principles outlined in the Declaration of Helsinki (Ethics Approval No. 14/9 October 2023).

All patients who came to the oral and maxillofacial surgery private clinic for treatment of impacted lower third molars and who met the inclusion criteria were encouraged to take part in the study. Eligibility for participation was determined based on the following criteria:

1. Stable general and oral health status;
2. Over 24 years old;
3. Fragilized mandibular angle by deep impacted molar, Pell and Gregory class II/IIIC impacted lower third molars [19] associated with pericoronitis or recurrent episodes of osteitis;
4. No temporomandibular pain/dysfunction, and no reduced mouth opening;
5. Consent to attend postoperative follow-up appointments.

Patients who did not meet the eligibility criteria were excluded in accordance with the criteria below:

1. Systemic diseases or receiving pharmacotherapy that contraindicates the surgical intervention;
2. Other Pell and Gregory classes of impacted lower third molars;
3. Current or recent acute episode;
4. Pregnant and breastfeeding mothers;
5. Patients with facial asymmetry;
6. Incapacity or unwillingness to adhere to requisite postoperative follow-up protocols.

In this cross-sectional study, out of a total of 117 patients with impacted lower third molars, 18 were selected after excluding those who did not meet the inclusion criteria (99 patients), resulting in a final convenience sample. All participants voluntarily provided written informed consent prior to their inclusion in the study.

### 2.2. Pre-Surgical Cone Beam Computed Tomography (CBCT)

**Figure 1:**
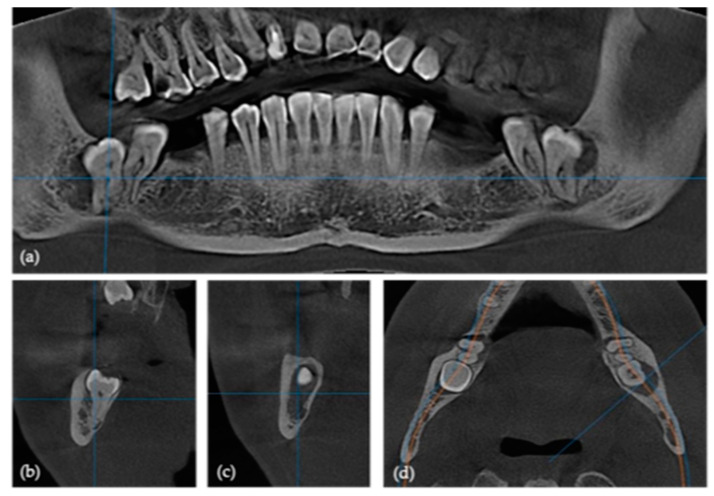
Preoperative CBCT images: (**a**) panoramic; (**b**,**c**) cross-sectional; and (**d**) axial views.

Before the surgical procedure, every subject underwent CBCT scanning. The CBCT images were acquired using SCANORA 3Dx (Soredex, Tuusula, Finland), operating under the parameters of a tube voltage of 60–90 kV, tube current of 4–10 mA, and a focal spot size of 0.5 mm (Figure 1).

**Figure 2:**
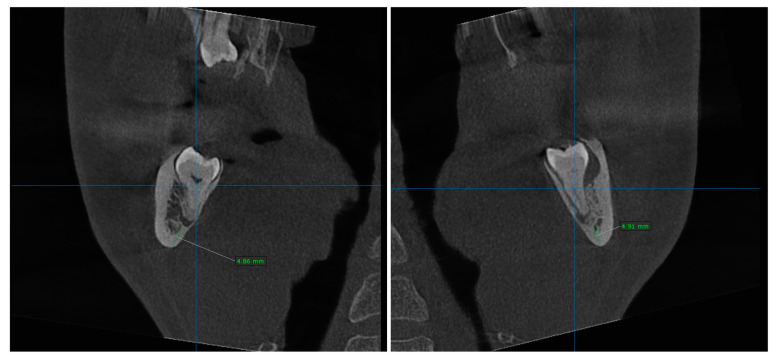
Cross-sectional views of deeply impacted mandibular third molars.

The images were analyzed to determine the angulation, depth, ramus distance, root/crown ratio, the surrounding bone, and the position of the inferior alveolar nerve (IAN), and to assess the surgical difficulty level of the extraction and the risk of mandibular fracture (Figure 2). A total of 26 mandibular third molars required this surgical intervention.

### 2.3. Design and Manufacturing of Customized Titanium Plates

**Figure 3:**
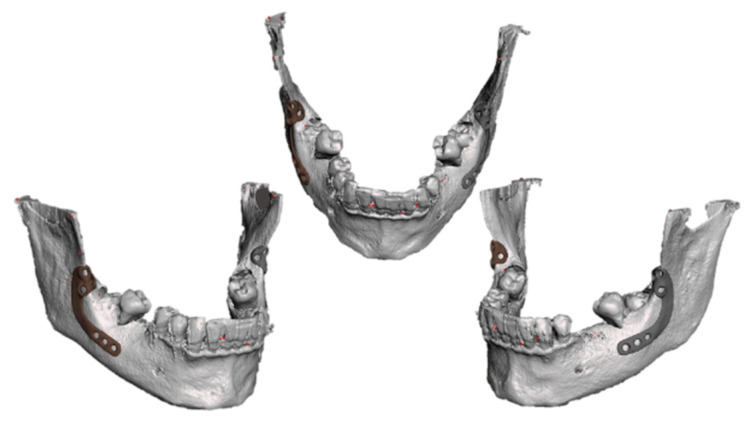
Virtual images of the designed customized plates.

We used the Exoplan 3.0 Galway software (Exocad GmbH, Darmstadt, Germany) to process the Digital Imaging and Communications in Medicine format (DICOM) data collected from the CBCT scans. This allowed us to design a customized titanium plate for each deeply impacted wisdom tooth and the drilling guides. The resulting model was saved as a standard tessellation (STL) file. The screw retention holes were precisely placed with relation to the adjacent dental roots and the inferior alveolar nerve (IAN), ensuring sufficient surgical space for performing ostectomy, odontotomy, and odontectomy procedures (Figure 3).

**Figure 4:**
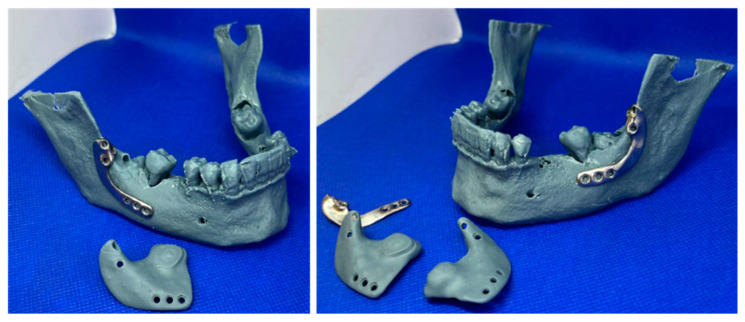
Three-dimensional printed drilling guides and customized titanium plates.

The final designs of the CTPs were prepared for manufacturing and printed using a Direct Metal Laser Sintering (DMLS) system, Mysint 100 (Sisma S.p.A., Piovene Rocchette, Italy), with titanium alloy powder, PowderRange Ti64 (Carpenter Technology Corporation, Philadelphia, PA, USA). Prior to packing and shipping, the CTPs underwent mechanical polishing on the buccal side, plasma cleaning, and sterilization. Additionally, the drilling guides were manufactured from acrylic resin (Phrozen Water-Washable Dental Model 3D Printer Resin, Phrozen Technology, Hsinchu, Taiwan) using an ASIGA 3D MAX UV printer (ASIGA, Alexandria, NSW, Australia) and then sterilized by immersion in peracetic acid for 25 min (Gigasept PAA, Schülke & Mayr GmbH, Norderstedt, Germany) (Figure 4).

### 2.4. Surgical Procedure

**Figure 5:**
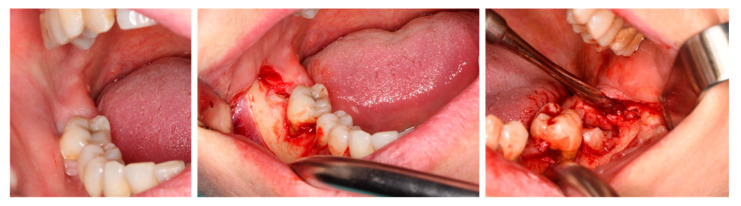
Intraoperative oral images: trapezoidal flap.

The patients’ general health was good, with blood tests within the normal range. At induction, the patients received an intravenous non-steroidal analgesic (Ketorol, Dr. Reddy’s Laboratories, Bucharest, Romania) and a loading dose of 2 g of amoxicillin. After rinsing for one minute with a 0.2% chlorhexidine solution (Curasept, Curasept S.p.A., Saronno, Italy), the surgical sites were cleaned with a sterile surgical drape. To minimize technical variables, the same operator performed all surgical procedures. An articaine solution (4%) with epinephrine (1:100,000; Ubistesin, 3M ESPE, Neuss, Germany) was used to induce IAN block anesthesia as well as buccal infiltration from the lower first molar (M1) to the retromolar area. The access involved a full-thickness trapezoidal flap from the lower M1 to the retromolar area, with reflection of the vertical ramus and external oblique line to facilitate the insertion and fitting of the plates (Figure 5).

**Figure 6:**
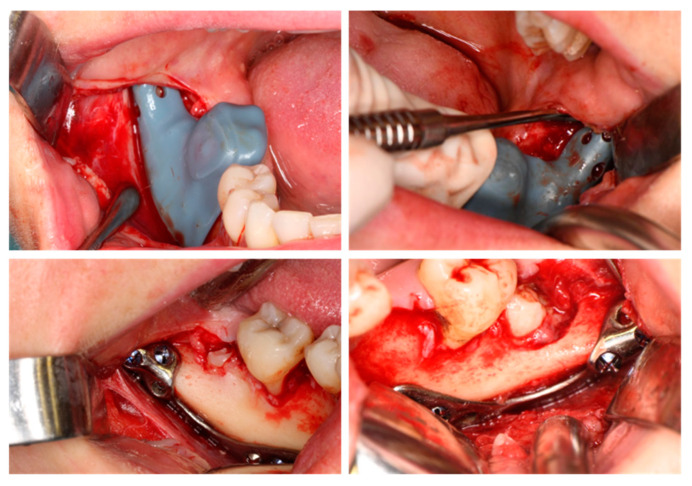
Intraoperative oral images: placing the CTPs.

After fitting, the CTPs were secured in place using 2/9 mm self-tapping screws with predrilling on the mandibular ramus, and 2/7 mm and 2/5.5 mm self-drilling screws on the distal site (Medicon eG, Tuttlingen, Germany). Once the plates were secured, a conservative ostectomy was performed using a low-speed straight handpiece and round burr under saline irrigation to expose sufficient parts of the crown for the odontectomy (Figure 6). The molar crowns were sectioned into one or more pieces to preserve as much bone as possible, followed by root separation when possible.

**Figure 7:**
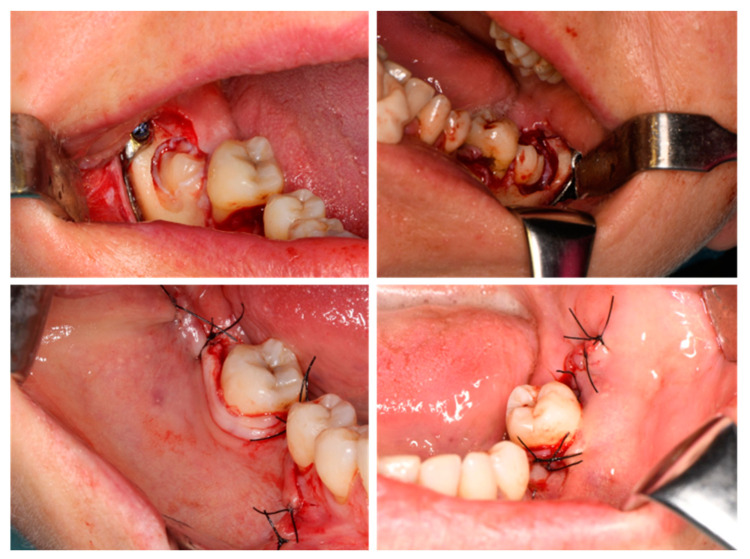
Intraoperative oral images: final step—suturing.

A cleavage plan between the bone and the roots is always recommended and, in our cases, this was achieved using a fine Lindemann burr. With a fine luxator placed in the cleavage plan and with the application of appropriate force, the roots were luxated and extracted. The post-extraction sockets were inspected for debris and to verify the presence and integrity of the IAN. The sockets were then filled with collagen sponges for both hemostatic and healing purposes. The customized titanium plates were designed to remain in place for three months to prevent delayed mandibular fracture, with the consideration that their removal would require a secondary surgical procedure. Suturing was performed with double (in the retromolar area) and simple sutures, using monofilament Supramid 4.0 (SMI, St. Vith, Belgium) (Figure 7).

**Figure 8:**
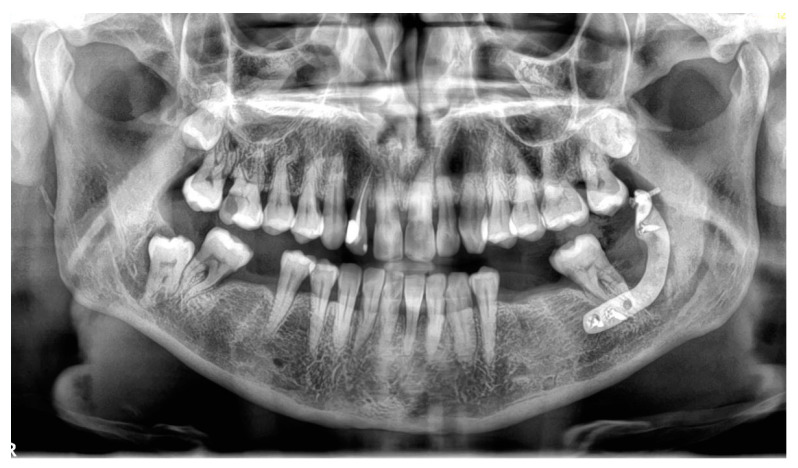
Post-surgical orthopantomography image.

Following the completion of the surgical procedure (Figure 8), a local infiltration of dexamethasone was administered to mitigate postoperative edema and discomfort [20]. Additionally, a 2 g dose of amoxicillin was prescribed for administration six hours postoperatively. Analgesics and anti-inflammatory medications were recommended for continued use over the following days to effectively manage pain and inflammation.

### 2.5. Anxiety, Pain, Swelling, Trismus, and Surgery Time Recording

To assess adult patients’ levels of dental anxiety, we used the shortened version of the Dental Anxiety Inventory (SDAI) [21]. The tool includes 9 statements rated on a 5-point scale from “ totally untrue “ to “ completely true,” with scores ranging from 9 to 45.

The nine statements [21] were as follows:

1. I become nervous when the dentist invites me to sit down in the chair.
2. When I know the dentist is going to extract a tooth, I am already afraid in the waiting room.
3. When I think of the sound of the drilling machine on my way to the dentist, I would rather go back.
4. I want to walk out of the waiting room the moment I think the dentist will not explain all the steps of the procedures to me.
5. As soon as the dentist gets their needle ready for the anesthetic, I shut my eyes tight.
6. In the waiting room, I sweat or freeze when I think of sitting down in the dentist’s chair.
7. On my way to the dentist, I get anxious at the thought that they will have to drill.
8. When I am sitting in the dentist’s chair not knowing what is going on in my mouth, I break in a cold sweat.
9. On my way to the dentist, the idea of being in the chair already makes me nervous.

The SDAI scores, recorded both before and one week after surgery, were categorized as follows: 9–10 indicating minimal dental anxiety, 11–19 mild anxiety in certain situations, 20–27 moderate anxiety with some ability to manage it, and 28–36 severe dental anxiety that could hinder regular treatment.

**Figure 9:**
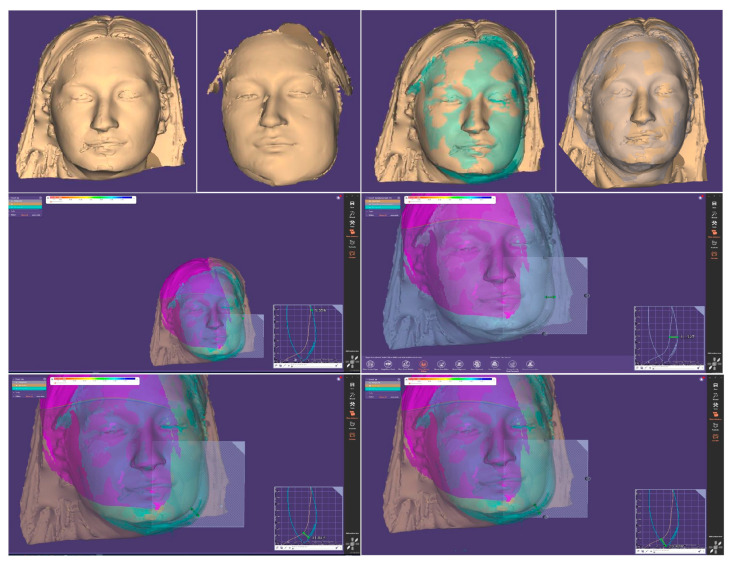
Three-dimensional facial scanning and superimposition for postoperative swelling analysis.

The edema was determined through preoperative (T0) facial scanning using the Range 3D Scanner (Revopoint, Shenzhen, China) at 72 h (T1) and 7 days (T2). The obtained images (in STL format) were imported into Exocad 3.0 Galway software (Exocad GmbH, Darmstadt, Germany) and T1-T0 and T2-T0 were superimposed. Measurements were performed using the cut view function (Figure 9).

Postoperative pain was evaluated using a 10-point visual analog scale (VAS), where 0 was equal to no pain and 10 denoted the highest level of pain imaginable.

Trismus was evaluated by assessing the maximum mouth opening, measured in millimeters as the distance between the incisal edges of the right upper and lower incisors using a caliper [12].

Pain, swelling, and trismus were recorded at three time points: prior to surgery (T0), three days post-surgery (T1), and seven days post-surgery (T2).

Surgery time was measured using a specialized chronometer, starting at the initial incision and ending when the suturing process began.

### 2.6. Statistical Analysis

Statistical analysis was conducted using IBM SPSS Statistics software (version 26.0 for Windows, SPSS Inc., Chicago, IL, USA). Descriptive statistics were summarized using frequency analysis. The normality of each variable was assessed using the Kolmogorov–Smirnov and Shapiro–Wilk tests. Group comparisons were performed using Mann–Whitney U and Kruskal–Wallis tests. Additionally, Pearson correlation was used to evaluate the relationships between outcome variables. A significant level of *p* ≤ 0.05 was established for all tests.

## 3. Results

The study included 8 males and 10 females, with ages ranging from 25 to 46 and an average age of 33.50 years (Table 1).

The dental anxiety levels of the study participants (N = 18) were assessed at baseline (T0) and one week after surgery (T2) using the SDAI scale. At T0, 38.9% of participants reported moderate anxiety, while at T2, the majority (44.4%) reported mild anxiety, reflecting a shift in anxiety levels. Although the average anxiety score decreased slightly from baseline, the difference was not statistically significant (*p* = 0.372). This suggests a trend toward reduced anxiety levels following surgery, but the changes were inconclusive (Table 2).

The outcome variables revealed an initial deterioration in clinical conditions, including facial swelling, pain, and trismus, immediately following surgery. However, all patients showed noticeable improvement one week postoperatively (Table 3 and Table 4).

Pearson’s correlation analysis revealed a moderate, statistically significant correlation among the first three measured variables, as well as a significant positive relationship between facial swelling in the submandibular measurements and the duration of surgery (*p* < 0.05). In contrast, no significant correlation was observed with the level of anxiety (Table 5).

## 4. Discussion

Mandibular third molar surgery is the most common surgical procedure in oral and maxillofacial surgery [22]. The etiological factors of impaction are generalized as systemic disorders and syndromes, and local factors such as a lack of space, odontoma, supernumerary teeth, odontogenic cysts and tumors, disruption in the dental follicle, and ankylosis [23]. The surgical extraction of deeply impacted mandibular third molars is a complex procedure that often involves excessive bone removal, potentially compromising the structural integrity of the mandible. In severe cases, this may result in immediate or delayed mandibular fractures. According to Krimmel et al. [24], mandibular fractures occurred an average of 14 days postoperatively, and the major risk factor seems to be advanced age and pre-existing bone lesions.

Our study aimed to evaluate a novel approach involving the use of a customized plate to reinforce the mandible during surgery and the recovery period, with the goal of minimizing the risk of angle fractures in cases involving Pell and Gregory class II/IIIC impacted lower third molars. These cases, often associated with pericoronitis or recurrent episodes of osteitis, were selected based on clinical experience, as they typically require extensive ostectomies to expose and remove deeply impacted wisdom teeth, leading to significant bone removal that can weaken the mandible and increase the risk of fractures both during and after surgery.

Cone beam computed tomography played a crucial role in accurately determining the spatial position of the impacted third molars and assessing bone volume. This information facilitated the integration of a CTP, which served as a preemptive osteosynthesis plate, reinforcing the mandible during and after surgery. Fabricated using 3D printing technology, the titanium plate was secured with 2 mm titanium screws, with three screws positioned on the distal side and two to three on the ramus. The plate’s design included a rough surface on the bone-contact side to enhance adherence, while the buccal side was finely polished for comfort and ease of placement. With a thickness of 1.5 mm, the plate featured a central curvature to avoid interfering with the surgical site, ensuring the smooth execution of the odontectomy.

According to some authors, the most frequent pathologies associated with mandibular wisdom tooth impaction are mandibular second molar carries or mandibular second molar periodontal pathology and pericoronitis [25].

Recent research demonstrated that the surgical extraction of impacted third molars frequently resulted in postoperative discomfort, swelling, and restricted mouth opening, significantly affecting patients’ health-related quality of life [26,27]. According to Yuasa et al. [28], postoperative discomfort and swelling were closely linked to the patient’s age and the complexity of the procedure. Additionally, previous studies highlighted the significant challenges associated with the extraction of deeply impacted mandibular third molars. These challenges included prolonged operative time and extensive osteoectomy impactions, which increased the risk of postoperative complications. According to some studies, postoperative pain begins once the local anesthetic affect has worn off and reaches its peak within 6 to 12 h after surgery. Postoperative edema gradually reaches its maximum by 48 h and regresses by the fourth day, with resolution 7 days after odontectomy [22,29].

Traditional methods for measuring facial swelling after surgical procedures, such as calipers, face bows, tape, and flexible rulers, have inherent limitations, providing only partial and inconsistent data [30]. These methods are also operator-dependent, reducing reliability and reproducibility. Advanced three-dimensional technologies offer an objective alternative [31,32,33]. In this study, facial swelling was recorded using non-invasive facial scanners and Exocad 3.0 Galway software at two postoperative time points, demonstrating the practicality of advanced digital tools in postoperative evaluations. Barone et al. [34] highlighted the effectiveness of facial scanners when integrated with 3DSlicer software, emphasizing their ability to deliver innovative and dependable analyses of postoperative edema. This combination allows for both qualitative and quantitative evaluations of facial swelling, including detailed measurements of linear and volumetric changes.

In this research, all interventions were performed by the same surgeon, with an average surgical time of 29.11 min, ranging from 19 to 38 min. The procedure involved the use of extensive trapezoidal flaps, extending from the mesial aspect of the lower first molar to the retromolar area, with some flaps reaching up to 1.5 cm vertically along the anterior border of the ramus to facilitate CTP insertion, fitting, and fixation. However, this innovative approach did not reduce the duration of the surgery; on the contrary, it led to an increase in operative time. The extensive periosteal elevation required for CTP insertion and proper fitting contributed to increased postoperative discomfort, including edema, pain, and trismus. Despite these initial complications, significant improvements were observed during recovery. Swelling significantly decreased from an average of 12.37 mm at 72 h post-surgery (T1) to 0.53 mm after one week (T2), with a *p*-value less of 0.001, indicating effective inflammation management. Pain levels, which peaked at T1 (mean score 4.89), significantly decreased to 1.11 by T2 (*p* < 0.001), reflecting successful postoperative pain control. Similarly, trismus improved significantly, with the mean mouth opening increasing from 29.28 mm at T1 to 41.46 mm at T2 (*p* < 0.001). All these findings led to the partial acceptance of the null hypothesis.

While the innovative approach using CTPs introduced certain challenges, several measures can be applied to minimize these limitations:

- minimally invasive flap design: Adaptations to the flap design could be explored to minimize periosteal elevation while maintaining adequate access for CTP insertion and fixation, for instance
- use of smaller, less extensive flaps could help reduce postoperative discomfort, including edema, pain, and trismus, without compromising surgical outcomes;
- use of advanced instruments: incorporating advanced surgical tools, such as ultrasonic bone cutters or piezoelectric devices, can enhance the precision and efficiency of the procedure while reducing trauma to the surrounding tissues;
- surgeon training and experience: surgeons with advanced training in CTP placement can perform the procedure more efficiently, reducing operative time and associated complications.
- postoperative care optimization: standardized postoperative care protocols, including the routine use of anti-inflammatory medications, analgesics, and cold compresses, can be increased with patient education.

This innovative approach offered significant benefits, including enhanced surgeon confidence and reduced stress and anxiety for both the surgeon and the patient. Additionally, regarding our study group, the absence of fractures highlighted the procedure’s effectiveness in maintaining structural integrity, supporting its potential to improve surgical outcomes and patient satisfaction in complex third molar extractions.

## 5. Conclusions

This study presented a novel method designed to reduce the risk of mandibular fractures during or following the surgical extraction of deeply impacted lower third molars. Importantly, no mandibular fractures were observed, indicating the effectiveness and safety of the technique. However, it should be noted that the approach remained operator-dependent, particularly in the phases of tooth exposure and odontectomy, as careful management of the pressure exerted on the residual bony structure was essential to ensure favorable surgical outcomes.

## Figures and Tables

**Table 1:** Characteristics of study participants (N = 18).

Variables		**N**	**%**
	Gender		
	Male	8	44.4
	Female	10	55.6
	Age (mean ± SD ^1^)	33.50 ± 6.913 ^1^	
	24 to 30 years	7	38.9
	Over 30 years	11	61.1
	Third molar	26 (100%)	
	3.8	11	42.3
	4.8	15	57.7
	Surgery time (min.)	29.11±5.593 ^1^ (minimum 19 min.-maximum 38 min.)	

^1^ Average and standard deviation.

**Table 2:** Dental anxiety scores of study participants (N = 18).

Dental Anxiety Score					
Anxiety	T0		T2		*p*-Value ^1^
	N	%	N	%	
Minimal	3	16.7	4	22.2	0.372
Mild	6	33.3	8	44.4	
Moderate	7	38.9	5	27.7	
Severe	2	11.1	1	5.7	
Scores	19.11 ± 6.986 ^1^ (minimum 9–maximum 31)		18.61 ± 6.572 ^1^ (minimum 9–maximum 30)		

^1^ Average and standard deviation; Mann–Whitney U test, *p* < 0.05.

**Table 3:** Facial swelling measurements at different time points.

Facial Swelling	T1-T0 [mm] Mean ± SD ^1^	T2-T0 [mm] Mean ± SD ^1^	*p*-Value ^1^
Temporo-zygomatic arch	0.48 ± 0.22	0.11 ± 0.041	<0.001
Mid-masseteric	10.40 ± 1.94	0.25 ± 0.035	
Gonial angle	11.09 ± 1.72	0.48 ± 0.048	
Submandibular	12.37 ± 1.83	0.53 ± 0.054	

^1^ Average and standard deviation; Mann–Whitney U test, *p* < 0.05.

**Table 4:** Pain and trismus at different time points.

Moment	Pain			Trismus		
	Mean ± SD ^1^	Minimum	Maximum	Mean ± SD ^1^	Minimum	Maximum
T0	2.39 ± 1.195	1	5	42.50 ± 4.46	36.13	50.51
T1	4.89 ± 1.367	3	8	29.28 ± 5.94	20.15	38.14
T2	1.11 ± 0.676	0	3	41.46 ± 4.58	33.64	49.62
*p*	<0.001			<0.001		

^1^ Average and Standard deviation; Kruskal–Wallis test, *p* < 0.05.

**Table 5:** Pearson’s correlation between surgery time and other variables.

		Surgery Time	
Facial swelling	Temporo-zygomatic arch	Pearson Correlation	0.565
		Sig. (2-tailed)	0.014 *
	Mid-masseteric	Pearson Correlation	0.561
		Sig. (2-tailed)	0.015 *
	Gonial angle	Pearson Correlation	0.542
		Sig. (2-tailed)	0.020 *
	Submandibular	Pearson Correlation	0.608
		Sig. (2-tailed)	0.007 *
Dental anxiety score		Pearson Correlation	0.264
		Sig. (2-tailed)	0.290

* Correlation is significant at the 0.05 level (2-tailed).

## Data Availability

The original contributions presented in the study are included in the article; further inquiries can be directed to the corresponding author.

## Abbreviations

The following abbreviations are used in this manuscript:

CBCT	Cone beam computed tomography
CTP	Customized titanium plate
DICOM	Digital imaging and communications in medicine
STL	Standard tessellation
DMLS	Direct metal laser sintering
IAN	Inferior alveolar nerve
SDAI	Shortened version of dental anxiety inventory
VAS	Visual analog scale
